# Differential Chromatin Accessibility, Gene Expression, and mRNA Splicing Between Developing Cochlear Inner and Outer Hair Cells

**DOI:** 10.1007/s10162-025-01005-z

**Published:** 2025-09-05

**Authors:** Chuan Zhi Foo, Anne Duggan, Elizabeth T. Bartom, Litao Tao, Jaime García-Añoveros

**Affiliations:** 1 Department of Anesthesiology, Northwestern University Feinberg School of Medicine, Chicago, IL 60611 USA; 2 Driskill Graduate Program, Northwestern University Feinberg School of Medicine, Chicago, IL 60611 USA; 3 Department of Biochemistry and Molecular Genetics, Northwestern University Feinberg School of Medicine, Chicago, IL 60611 USA; 4 Department of Biomedical Sciences, Creighton University, Omaha, NE 68178 USA; 5 Departments of Neuroscience and Neurology, Northwestern University Feinberg School of Medicine, Chicago, IL 60611 USA; 6 Hugh Knowles Center for Clinical and Basic Science in Hearing and Its Disorders, Northwestern University, Chicago, IL USA

**Keywords:** Inner hair cell (IHC), Outer hair cell (OHC), RNA-seq, ATAC-seq, Alternative mRNA splicing, Novel promoters

## Abstract

**Purpose:**

The mammalian cochlea has two types of low abundance and highly specialized inner (IHC) and outer (OHC) mechanosensory hair cells. Their malfunction or death is a common cause of congenital and acquired deafness. IHCs and OHCs exhibit different transcriptomes during development. We wondered how differences in gene expression are regulated at the chromatin level in developing IHCs and OHCs, and whether there were also differences in mRNA splicing between IHCs and OHCs.

**Methods:**

We separately collected developing mouse IHCs and OHCs to identify their mRNAs and chromatin states. We examined their transcriptomes by bulk (full coverage) RNA-seq from six biological replicates each to reveal differences in gene expression and in alternative mRNA splicing. We also examined their chromatin conformation by bulk ATAC-seq from two biological replicates each to reveal open vs. closed promoter and enhancer elements. We then compared ATAC-seq with RNA-seq datasets to determine if differential chromatin accessibility can account for differential gene expression. Each biological replicate consists of hair cells pooled from multiple neonatal mice of both sexes.

**Results:**

We found that developing IHCs and OHCs have differentially accessible promoters in many differentially expressed genes. This includes functional genes whose expression is incipient in neonatal hair cells but will be maintained throughout life, and developmental genes which are only expressed transiently. We also found that different mRNA isoforms result from alternative mRNA splicing and transcription start sites. Finally, our data reveals that cochlear hair cells utilize unique promoters and mRNA isoforms absent in other cell types.

**Conclusion:**

Differential transcriptomes between developing hair cell types result from pre- and post-transcriptional mechanisms. The unique promoters and mRNA isoforms in cochlear HCs highlight the importance of elucidating transcriptomes and epigenomes of rare cell types. We provide a comprehensive resource for the identification of promoters and mRNA isoforms of genes expressed by neonatal IHCs or OHCs, which is publicly-accessible for visualization of any gene of interest at https://igvviewer.s3.us-east-2.amazonaws.com/index.html.

**Supplementary Information:**

The online version contains supplementary material available at 10.1007/s10162-025-01005-z.

## Introduction

The mammalian cochlea has two types of mechanosensors, inner (IHC) and outer (OHC) hair cells, which exhibit different biological properties. IHCs, endowed with a prominent pre-synaptic apparatus, are the main sensory receptors that transmit signals to the brain. OHCs, capable of a unique mode of electromotility, are primarily responsible for mechanical amplification to enhance sensitivity and tuning. In addition, IHCs are primarily pre-synaptic and densely innervated by afferent neurons, while OHCs are largely post-synaptic and mainly innervated by efferents.

Hair cells (HCs) develop from the prosensory domain of the otic vesicle, which in turn derives from the otic placode (non-neural ectoderm). During development, cells of the cochlear sensory epithelium initially express *Sox2* [1], but this later is lost in hair cell precursors, while retained in supporting cells (SCs) [2]. Hair cell precursors then upregulate *Atoh1*, followed by *Pou4f3* and finally *Gfi1* [3–5]. In the absence of ATOH1, hair cells do not form [3], while in the absence of POU4F3 or GFI1, hair cells form but fail to mature properly and eventually die [4, 6].

*Insm1* is expressed in OHCs shortly after *Atoh1* [6], but its expression subsides a few days after birth. This is then followed by the expression of *Ikzf2*, whose expression in OHCs is maintained for life [7]. In the absence of INSM1, about half of the OHCs transdifferentiate into IHCs, and animals exhibit hearing impairment consistent with OHC dysfunction [8]. Early in transdifferentiation, a subset of IHC-enriched genes is upregulated in OHCs, among them *Tbx2* [8, 9]. *Tbx2* was found to be a master regulator of IHC differentiation, as all IHCs transdifferentiated into OHCs in the absence of TBX2, while OHCs that ectopically expressed TBX2 convert into IHCs [9], a result confirmed by various approaches [10, 11].

We previously found with RNA-seq that neonatal (P0) IHCs and OHCs had different developmentally transient gene expression profiles. The genes whose expression was enriched in IHCs relative to OHCs and vice versa at P0 had little overlap with the corresponding sets of genes in adults [8, 12], suggesting that transcriptomic differences underlie the differential development of each cell type.

Here we wondered whether the transcriptional differences between IHCs and OHCs, either transient or permanent, may be due to differential chromatin accessibility of promoters or enhancers. We also examined whether posttranscriptional processes such as alternative mRNA splicing further contribute to the differential transcriptomes of IHCs and OHCs. Our examination reveals the unique mRNA isoforms and promoters of each gene used by IHCs and/or OHCs.

## Results

### Some IHC- and OHC-Enriched Genes at P0 Are Hair Cell Specific, While Others Are Co-expressed by Other Cochlear Cell Types

To begin with, we re-examined our previously-published bulk RNA-seq data [8] obtained from separate pools of IHCs and OHCs using the latest release of the mouse genome (mm39), so as to use an updated transcriptome as the base for the subsequent analysis of promoters and enhancers. Reads were aligned to the genome using STAR [13]. The number of reads per gene was counted with HTSeq using the intersection-nonempty mode to resolve reads overlapping more than one feature [14, 15]. Differential gene expression was then computed using edgeR [16–19], which is generally more stringent than DESeq2, in order to decrease the potential number of false positives, in which we considered the genes whose false discovery rate-adjusted *p*-value (FDR) is less an 0.05 as being significantly differentially expressed. Moreover, the standard edgeR workflow also filters out the genes whose expression is low across all samples, further reducing the number of potential false positives due to noise. With this more stringent and updated approach, we found that 752 genes were enriched in IHCs (Table S1) and 531 genes in OHCs (Table S2), as compared to the 938 IHC- and 691 OHC-enriched genes previously estimated using DESeq2 and older genome versions [8]. This updated approach results in the detection of an additional 297 IHC- and 288 OHC-enriched genes but excludes 115 and 137 genes previously deemed to be IHC- and OHC-enriched, respectively. However, as far as we know, none of the genes now excluded has known roles in hair cells (HCs). On the other hand, the newly identified genes include the OHC-enriched *Prox1*, known to be expressed and perhaps function in developing outer hair and supporting cells [20].

We next sought to identify which of the genes preferentially expressed in either IHCs or OHCs were exclusive to hair cells and which were also expressed in other cochlear types, such as supporting or mesenchymal cells. Our strategy was to compare our IHC- and OHC-enriched transcriptomes with those of hair cells vs other cochlear cells publicly available. Previously, Cai et al. [21] had dissociated cochlear cells (including organs of Corti and adjacent tissues) at P0 and sorted apart GFP-labelled hair cells from unlabeled non-hair cells, then performed RNA-seq. Using TPMCalculator [22], we computed the transcripts per million (TPM) values, which are the number of mapping reads normalized for gene length and the total number of reads [23] for each gene in HCs and non-HCs (Table S3).

We chose the thresholds for a gene to be considered expressed in HCs and not in other cells by examining individual genes with known expression patterns (mostly revealed by in situ hybridization or immunohistochemistry) and chose a TPM of 5 for IHC-enriched genes, and a TPM of 1 for OHC-enriched genes. Based on published literature, our criteria confirmed that the IHC-enriched *Otof* [24], *Chrna1*, *Chrng* [25], *Kif21b* [21], *Cabp2* [26], *Gfi1* [20], and *Ldb3* [27] were exclusively expressed in HCs, while *Fgf10* [28] and *S100a1* [29] were co-expressed in IHCs and some other cochlear non-hair cell types. Likewise, we confirmed *Bmp2* [30] to be OHC-specific and *Robo2* [31], *Efna5* [32], and *Prox1* [20] not to be HC-specific.

**Fig. 1:**
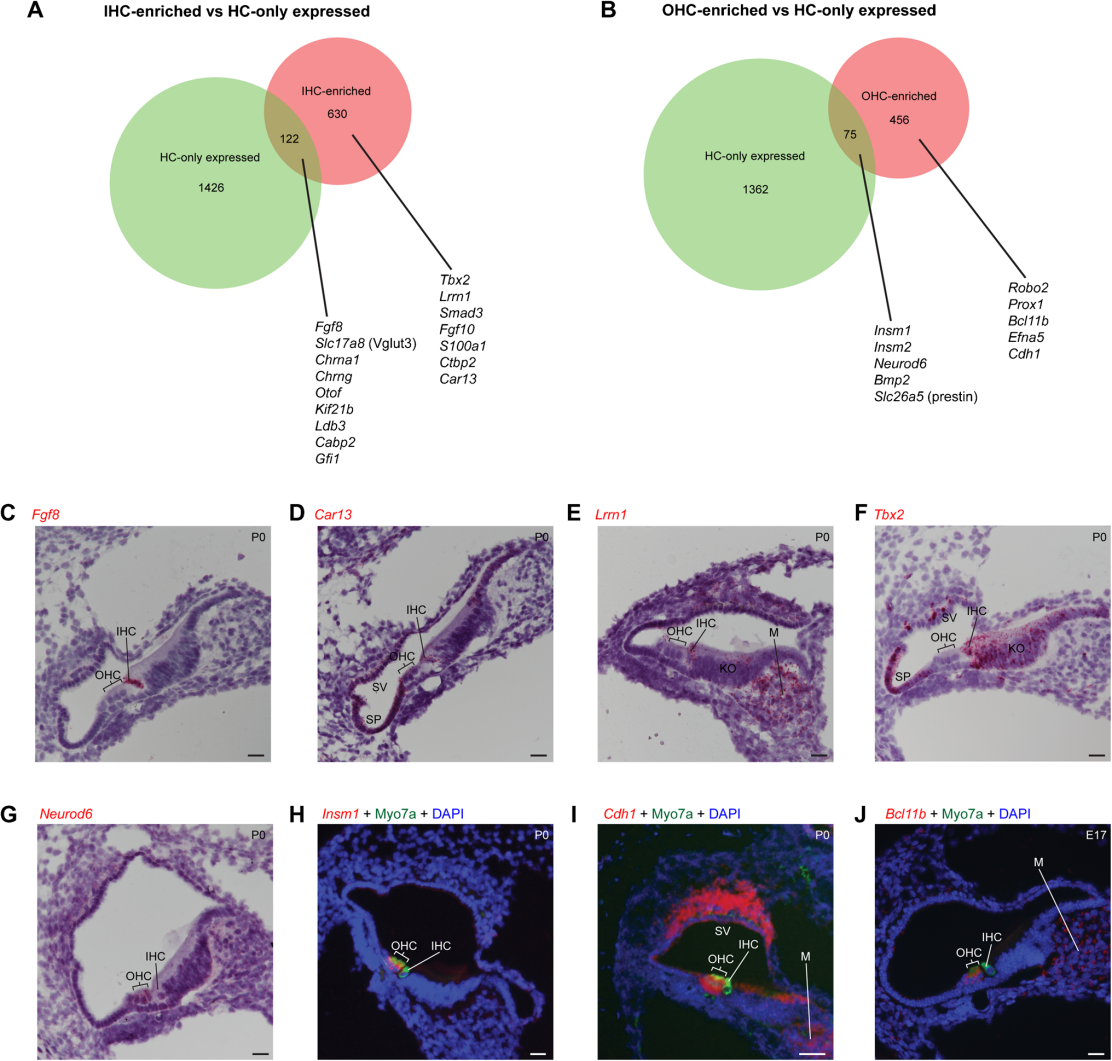
Some IHC- and OHC-enriched genes are unique to HCs, and others are also expressed in other cochlear cells, including some that are compartment specific. **A** Overlap of IHC-enriched genes (red) and genes that are only expressed in HCs (green), with a threshold of TPM > 5 taken to mean that the gene is expressed. Listed are examples of genes that are exclusively expressed in IHCs (overlapping) or that are also expressed in other cochlear cell types (non-overlapping). Related to Tables S4 and S5. **B **Overlap of OHC-enriched genes (red) and genes that are only expressed in HCs (green), with a threshold of TPM > 1 taken to mean that the gene is expressed. Listed are examples of genes that are exclusively expressed in OHCs (overlapping) or that are also expressed in other cochlear cell types (non-overlapping). Related to Tables S6 and S7. **C**–**J** RNA in situ hybridization images of various genes, taken with cross sections of the cochlea. Lateral is to the left and medial to the right. Scale bar is 20 µm. Age of the mouse is indicated on the top right of each image. We previously showed a portion of panel D restricted to the organ of Corti in Wiwatpanit et al. [8]. Cell types or domains of the cochlea are indicated as follows: *IHC*, inner hair cell; *OHC*, outer hair cell; *SP*, spiral prominence; *SV*, stria vascularis; *KO*, Kölliker’s organ; *M*, mesenchymal cells

We also performed RNA in situ hybridization to further validate our classification and confirmed that of the IHC-enriched genes, *Fgf8* was indeed specific to IHCs (Fig. 1C), while *Car13*, *Lrrn1*, and *Tbx2* were expressed by IHCs and other cells of the cochlea (Fig. 1D–F). Likewise, of the OHC-enriched genes, *Neurod6* and *Insm1* were confirmed to be OHC-specific (Fig. 1G–H) whereas *Cdh1* and *Bcl11b* were not: *Cdh1* is expressed in both outer hair and supporting cells, and *Bcl11b* is expressed in OHCs and cochlear mesenchymal cells (Fig. 1I–J).

Altogether our analysis revealed that 122 out of 752 IHC-enriched genes (Fig. 1A, Tables S4, S5) and 75 of 531 OHC-enriched genes (Fig. 1B, Tables S6, S7) are exclusively expressed in HCs, while the remaining 630 and 456 were deemed to be also expressed in other cochlear cell types. In combination with our examination of open vs. closed chromatin (described later in this manuscript), these results will be useful in identifying promoter elements that may selectively drive expression solely in IHCs or OHCs.

### IHCs and OHCs Exhibit Differential Splicing at P0

**Fig. 2:**
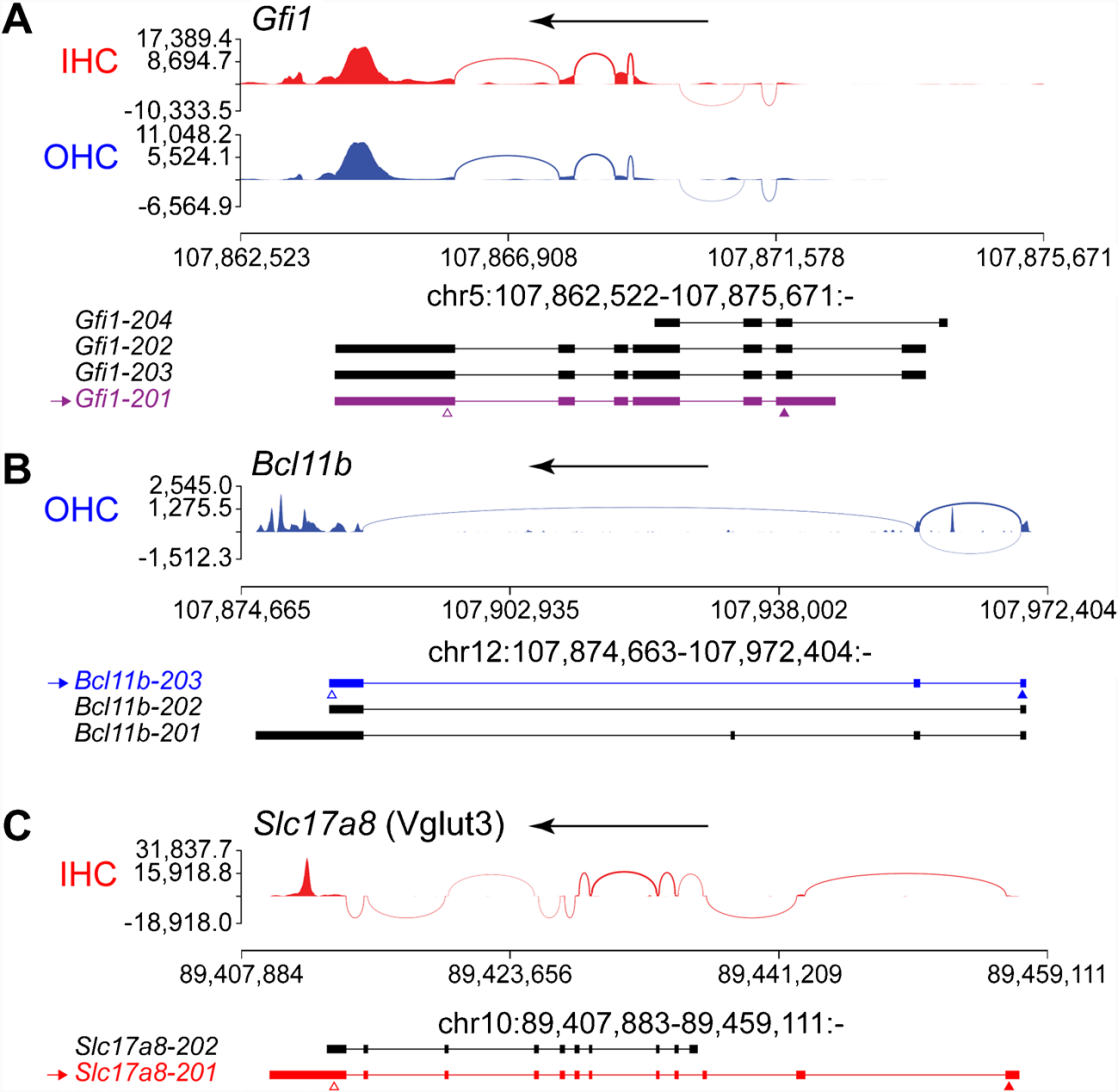
Bulk RNA-seq can be used to identify the mRNA isoform of genes expressed by HCs. Sashimi plots revealing the exons (peaks) and spliced introns (curved lines linking exons) that generate hair cell-specific (red for IHCs and blue for OHCs) mRNA isoforms. Top arrows point to the direction of gene transcription. Below each plot are depicted the known mRNA isoforms (exons thick, and introns thin, lines) produced by that gene. The transcript isoform of gene expressed in both types of HCs (purple), OHCs (blue), or IHCs (red) is indicated with an arrow to the left. Transcripts not expressed by HCs are in black. Arrowheads point to the start (filled) and end (open) of the coding sequences. Arrow at the top of each plot indicates direction of gene. Sashimi plots revealing the exons (peaks) and spliced introns (curved lines linking exons) that generate hair cell-specific (red for IHCs and blue for OHCs) mRNA isoforms. Top arrows point to the direction of gene transcription. Below each plot are depicted the known mRNA isoforms (exons thick, and introns thin, lines) produced by that gene. The transcript isoform of gene expressed in both types of HCs (purple), OHCs (blue), or IHCs (red) is indicated with an arrow to the left. Transcripts not expressed by HCs are in black. Arrowheads point to the start (filled) and end (open) of the coding sequences. Arrow at the top of each plot indicates direction of gene. **A** Sashimi plot of *Gfi1* in IHCs and OHCs. **B** Sashimi plot of *Bcl11b*, an OHC-specific gene, in OHCs. **C** Sashimi plot of *Slc17a8* (Vglut3), an IHC-specific gene, in IHCs

In addition to differences in gene expression, differences between IHCs and OHCs might also be due to differential mRNA splicing. Unlike microarrays or the most common 3′ end-based Library synthesis methods such as the one used by 10x Genomics for single-cell RNA-seq, bulk RNA-seq allows us to perform splicing analysis for identifying the mRNA isoforms present in hair cells. We first individually inspected some genes that are expressed in HCs and can produce multiple alternatively spliced RNAs in order to identify the ones expressed by HCs. We found that in HCs *Gfi1* expresses the shorter isoform lacking the first exon and with a downstream translation start codon (Fig. 2A). We also found that the isoform of *Bcl11b* expressed in OHCs is the one lacking the third exon (Fig. 2B), while the isoform of *Slc17a8* (Vglut3) expressed in IHCs is the full-length one (Fig. 2C).

**Fig. 3:**
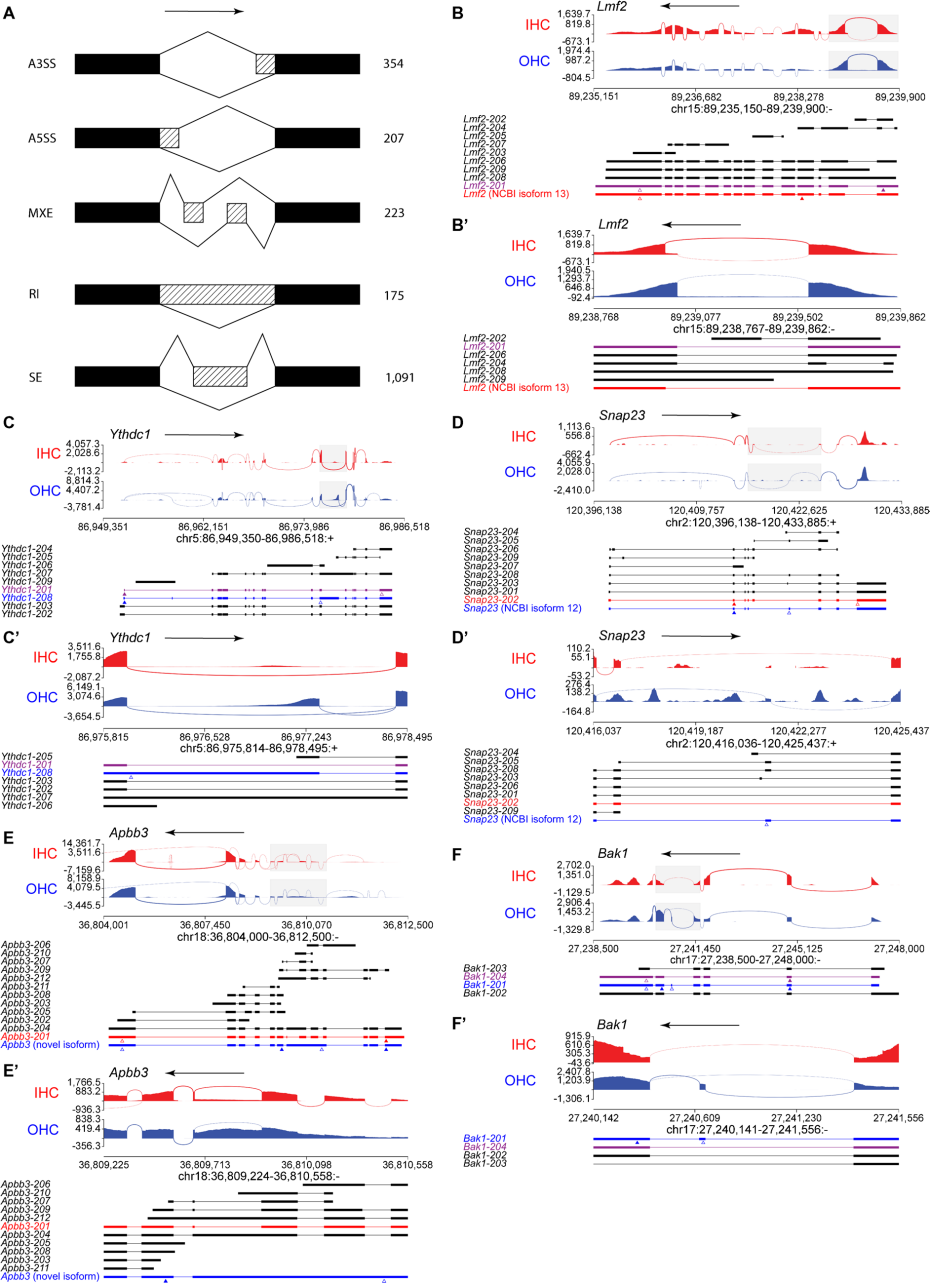
IHCs and OHCs exhibit differential splicing at P0. **A** Schematic of different types of differential splice events detected by rMATs: alternative 3′ splice site (A3SS), alternative 5′ splice site (A5SS), mutually exclusive exons (MXE), retained intron (RI), and skipped exon (SE). Numbers on right indicate the number of statistically significant differential splice events (FDR < 0.05, ΔPSI ≥ 10%) of each type detected by rMATS. **B-F’** Sashimi plots revealing the exons (peaks) and spliced introns (curved lines linking exons) that generate hair cell-specific (red for IHCs and blue for OHCs) mRNA isoforms. Top arrows point to the direction of gene transcription. Below each plot are depicted the known mRNA isoforms (exons thick, and introns thin, lines) produced by that gene. The transcript isoform of gene expressed in both types of HCs (purple), OHCs (blue) or IHCs (red) are indicated with an arrow to the left. Transcripts not expressed by HCs are in black. Arrowheads point to the start (filled) and end (open) of the coding sequences. Arrow at the top of each plot indicates direction of gene. **B**, **B’** Example of A3SS (Lipase Maturation Factor 2 [*Lmf2*]), with full length of gene in **B** and the area in the box zoomed in in **B’**. The IHC isoform is not present in Ensembl’s database but can be found in NCBI’s database. Related to Table S8. **C**, **C’** Example of A5SS (YTH N6-Methyladenosine RNA Binding Protein C1 [*Ythdc1*]), with full length of gene in **C** and the area in the box zoomed in in **C’**. Related to Table S9. **D**, **D’** Example of mutually exclusive exons (Synaptosome Associated Protein 23 [*Snap23*]) with full length of gene in **D** and area in the box zoomed in in **D’**. The OHC isoform is not present in Ensembl’s database but has been found in NCBI’s database. Related to Table S10.. **E**, **E’** Example of retained introns (Amyloid Beta Precursor Protein Binding Family B Member 3 [*Apbb3*]) with full length of genes in **E**, and area in the box zoomed in in **E’**. Related to Table S11. **F**, **F’** Example of spliced exon (BCL2 Antagonist/Killer 1 [*Bak1*]), with full length of genes in **F** and area in the box zoomed in in **F’**. Related to Table S12

We then proceeded to search for differential splicing on a genome-wide scale using rMATS [33–36], which uses an event-based approach to determine if there is differential splicing between IHCs and OHCs in five distinct categories: alternative 3′ splice site (A3SS), alternative 5′ splice site (A5SS), mutually exclusive exon (MXE), retained intron (RI), and skipped exon (SE). For this study, we consider splice differences with FDR < 0.05 and an inclusion level difference of at least 0.1 (i.e., difference in percent spliced in (ΔPSI) of at least 10%) to be significant. We found a total of 2396 differential splice events in 1406 genes between IHCs and OHCs, of which there are 354 A3SS in 313 genes, 207 A5SS in 191 genes, 223 MXEs in 174 genes, 175 RIs in 165 genes, and 1091 SEs in 818 genes (Fig. 3A, Tables S8- S12).

An example of an A3SS can be seen in the gene Lipase Maturation Factor 2 (*Lmf2*), which is believed to be a transmembrane protein that is involved in protein maturation, based on its apparent homology with Lipase Maturation Factor 1 (*Lmf1*) [37]. In IHCs, there is a shorter isoform, not present in OHCs, which uses a downstream splice acceptor site for Exon 4. This transcript has a truncated open reading frame, with the start codon in Exon 4 instead of Exon 1 (Fig. 3B, B’).

An example of an A5SS can be seen in the gene YTH N6-Methyladenosine RNA Binding Protein C1 (*Ythdc1*), which is involved in the export of N6-methyladenosine methylated mRNAs from the nucleus [38], and can also bind to methylated adenines in DNA [39]. Of this gene, OHCs exhibit an isoform with a downstream splice donor site for Exon 12, resulting in a longer transcript that introduces premature stop codons in all reading frames in the said exon, and is predicted by NCBI (National Center for Biotechnology Information) to function as a non-coding RNA (Fig. 3C, C’).

An example of a MXE can be found in the gene Synaptosome Associated Protein 23 (*Snap23*), which facilitates the fusion of vesicles to the plasma membrane [40, 41]. The transcript seen to be expressed in IHC appears to be isoform 202 in Ensembl’s database, whose fifth exon does not appear to be expressed in OHCs (Fig. 3D, D’). On the other hand, the fifth exon of the OHC isoform appears to use an alternative downstream exon that introduces a premature stop codon and is predicted to function as a non-coding RNA by NCBI.

An example of a RI can be found in the gene Amyloid Beta Precursor Protein Binding Family B Member 3 (*Apbb3*), which interacts with the intracellular domain of Alzheimer's *β*-amyloid precursor protein (APP) [42], and facilitates the secretion of *β*-amyloid peptide [43]. OHCs exhibit a novel isoform of this mRNA that retains the introns 4–6 (spliced in the IHC isoform), resulting in premature stop codons, though there is a downstream start codon and open reading frame from the retained introns (Fig. 3E, E’). This novel isoform has not been characterized, but it could produce a truncated and novel protein or function as a non-coding RNA.

An example of a SE can be found in the gene BCL2 Antagonist/Killer 1 (*Bak1*) that localizes to the mitochondria and is involved in inducing apoptosis [44, 45] and necrosis [46]. OHCs express an isoform of the gene that includes Exon 5, which is skipped in the other isoform, the one present in both IHCs and OHCs (Fig. 3F, F’). This introduces a premature stop codon in Exon 5 and is predicted by NCBI to function as a non-coding RNA, though there is also a possibility that a truncated protein is produced due to the presence of a downstream start codon and open reading frame in the penultimate exon.

While here we only describe a few examples, our database may be examined to identify the mRNA isoforms of any given gene expressed in IHCs, OHCs, or both.

### Neonatal IHCs and OHCs Have Differential Chromatin Accessibility

To reveal differences in chromatin accessibility between IHCs and OHCs, we performed assay for transposase-accessible chromatin using sequencing (ATAC-seq) on IHCs and OHCs. We first obtained organs of Corti from neonates (P0), dissociated them, and collected pools of IHCs and OHCs by fluorescence-activated cell sorting (FACS), using the same genetic strategy we had previously employed for RNA-seq [8]. For the first batch of cells, we pooled 3014 IHCs and 12,685 OHCs from 9 pups from 2 Litters. For the second batch of cells, we pooled 2932 IHCs and 11,706 OHCs from 9 pups from 3 litters. Following cell sorting, we performed library synthesis on the separate IHC and OHC pools using a modified protocol based on what had previously been published by the Greenleaf lab [47, 48].

Libraries were sequenced using the Illumina NextSeq 500 sequencer. Reads were aligned to the mm39 mouse genome using Bowtie 2 [49, 50]. ATAC-seq peaks, which represent regions of accessible chromatin, were identified using Genrich [51]. The number of reads under each peak was quantified using HOMER’s annotatePeaks.pl pipeline [52]. Differential accessibility of chromatin between IHCs and OHCs under each peak was computed using the R package edgeR [16–19].

**Fig. 4:**
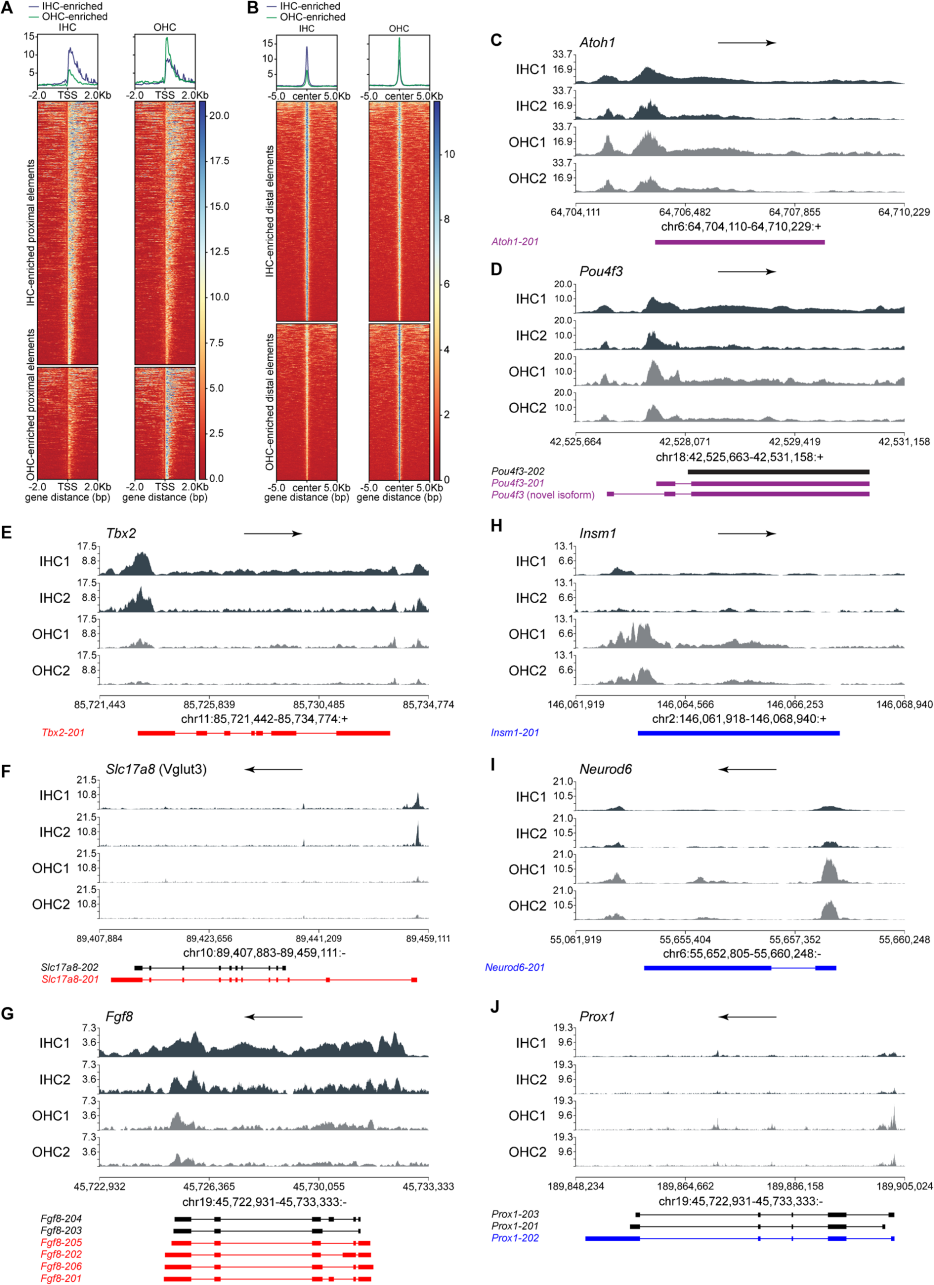
ATAC-seq reveals accessible proximal and distal chromatin elements in IHCs and OHCs, including differentially accessible proximal elements for some differentially expressed genes. Related to Tables S14 and S15. **A** Heat map of 1050 IHC-enriched (top) and 553 OHC-enriched (bottom) proximal elements. The Line graph at the top represents read coverage in arbitrary units, with IHCs in blue and OHCs in green. In the heat maps below, blue and red indicate high and lower read coverage, respectively. The heat maps are centered around the transcription start site, with a width of 2 kb on each side. **B** Heat map of 2877 IHC-enriched enhancers (top) and 1899 OHC-enriched enhancers (bottom). The Line graph at top represents read coverage in arbitrary units, with IHCs in blue and OHCs in green. Blue indicates higher read coverage and red indicates lower read coverage in arbitrary units. Heat maps are centered around the center of the peak, with a width of 5 kb on each side. **C**–**J** ATAC-seq traces of gene loci from IHC (black) and OHC (grey) chromatin. Below each plot are depicted the known mRNA isoforms (exons thick, and introns thin, lines) produced by that gene. The transcript isoform of the gene expressed in both types of HCs (purple), OHCs (blue), or IHCs (red) is indicated with an arrow to the left. Transcripts not expressed by HCs are in black. Arrow at the top of each plot indicates the direction of the gene. **C**, **D** Genes with no significant differences in chromatin state: **C** *Atoh1* and **D** *Pou4f3*. **E**–**G** Genes with more open chromatin in IHCs: *Tbx2* (**E**), *Slc17a8* (Vglut3) (**F**), and *Fgf8* (**G**). **H**–**J** Genes with more open chromatin in OHCs: **H** *Insm1*, **I** *Neurod6*, and **J** *Prox1*

For this study, we consider proximal elements to be within 2 kb of the nearest transcription start site, representing potential promoters, and distal elements to be from 2 to 200 kb of the nearest transcription start site, representing potential enhancers, as previously defined by Tao et al. [53]. To separate the peaks into the two categories, we first downloaded a list of transcription start sites from UCSC Genome Browser’s Table Browser [54], using the chromStart field as the transcription start site (TSS) for forward-oriented genes and the chromEnd field as the TSS for reverse-oriented genes. We then used the Bedtools Window [55] function to categorize the IHC- and OHC-enriched peaks based on their distance from the nearest TSS. Out of 61,814 peaks in IHCs and OHCs, 3348 were enriched in IHCs (Table S13) and 2085 in OHCs (Table S14). Of the IHC-enriched peaks, 1050 (31.4%) were proximal elements (Fig. 4A, Table S15) and 2272 (67.9%) distal elements (Fig. 4B, Table S16), while of the OHC-enriched peaks, 446 (21.4%) were proximal (Fig. 4A, Table S17) and 1615 (77.5%) distal elements (Fig. 4B, Table S18).

We then proceeded to visually examine the ATAC-seq plots of some genes implicated in HC function and development. We found that *Atoh1* and *Pou4f3*, two genes that are critical for HC development, had open promoter regions (Fig. 4C–D). Neither of these genes is differentially expressed between IHCs and OHCs, and their ATAC-seq peaks also did not reveal significant differences in chromatin accessibility. We then examined the genes *Tbx2*, *Slc17a8* (Vglut3), and *Fgf8*, whose expression was previously described as enriched in IHCs relative to OHCs, and found that their promoter regions were more accessible in IHCs than in OHCs (Fig. 4E–G). In the case of *Fgf8*, it appeared that it was not just the promoter region but the entire length of the gene that was more open in IHCs. We then examined *Insm1*, *Neurod6*, and *Prox1*, whose expression had previously been shown to be enriched in OHCs relative to IHCs. Similarly, their promoter regions were more open in OHCs than in IHCs (Fig. 4H–J).

We next looked for ATAC-seq peaks that were enriched in HCs relative to SCs and vice versa by re-analyzing prior data from Tao et al. [53]. They had collected HCs and SCs from P1 mice, using an *Atoh1*-GFP marker to label HCs, and using the *Lnfg*-GFP marker that labels inner border cells (IBCs), inner phalangeal cells (IPhCs), inner pillar cells (IPCs), and outer pillar cells (OPCs) and Deiters’ cells (DCs) to allow their collection by flow cytometry. In order to distinguish peaks (potential regulatory elements such as promoters or enhancers) that may regulate gene expression solely in hair cells from those that may regulate expression in groups of cell types (such as the inner or outer compartments of the organ of Corti), we used Bedtools to determine if the IHC- or OHC-enriched peaks at P0 from our data overlapped with the HC- or SC-enriched peaks at P1. Out of 1050 proximal elements that were enriched in IHCs (vs OHCs) at P0, 138 were enriched in HCs (vs SCs) (Table 1, Table S19) at P1 and 912 were not (Table 1, Table S20). Out of 446 proximal elements that were enriched in OHCs at P0, 77 were enriched in HCs at P1 (Table 2, Table S21), and 369 were not (Table 2, Table S22). IHC-enriched proximal elements that were enriched in HCs included the promoter regions of some genes known to be HC-specific (and preferentially in IHCs) such as *Gfi1* and *Pvalb*, while OHC-enriched proximal elements that were enriched in HCs included promoter regions of some genes known to be exclusively expressed in OHCs but not SCs, such as *Insm1* and *Bcl11b*. Of note, *Bcl11b* is expressed in mesenchymal cells of the organ of Corti, but within the sensory epithelium, it is exclusively expressed in OHCs (Fig. 1J).

**Table 1:** IHC-enriched ATAC-seq proximal elements ranked by FDR-adjusted *p*-values that are enriched or not enriched in HCs relative to SCs. Related to Tables S19 and S20

IHC-enriched proximal elements	
HC-enriched	Not HC-enriched
Cd164l2	Gm43584
Kcnip3	Tbx2
Rgl1	Cubn
Shtn1	Lrrc30
P2rx5	Siva1
Rnf157	Rcc1l
Gm4791	Slc17a8
Trim71	Gtpbp1
Coil	Gm28845
ENSMUSG00002076614	Gm50277
Ctbp2	Maff
St8sia3	Dffa
Ecel1	Synj2
Ranbp9	Gm35409
Ctbp2	Lrrc3c
A330094K24Rik	Tmem132c
Cys1	Gm9195
A530016L24Rik	Smad3
Stk32c	Rdm1
Gpr153	Tmem274

**Table 2:** OHC-enriched ATAC-seq proximal elements ranked by FDR-adjusted *p*-value that are enriched or not enriched in HCs relative to SCs. Related to Tables S21 and S22

OHC-enriched proximal elements	
HC-enriched	Not HC-enriched
Insm1	Efna5
B3galt5	Zfp503
Mia3	Pold1
Agpat2	Tmem178b
Col9a2	Cyp2j12
Bcl11b	Arrb2
Zfp804a	Gm26604
Tmem163	Unkl
Tlcd3b	Atxn7l2
Gm23819	2510002D24Rik
C630004L07Rik	Neurod6
ENSMUSG00002076905	Krtap15
Grip2	G630018N14Rik
Gse1	Dnaja3
Krtap16-1	Timm50
Fgfr3	Gm24836
Fam20c	Gm36543
Gm35963	Gm26971
1700092K14Rik	Gpsm1
Gm13096	Tac4

### Most Genes Highly Expressed in Neonatal IHCs or OHCs Have Accessible Proximal Elements (Likely Promoters)

**Fig. 5:**
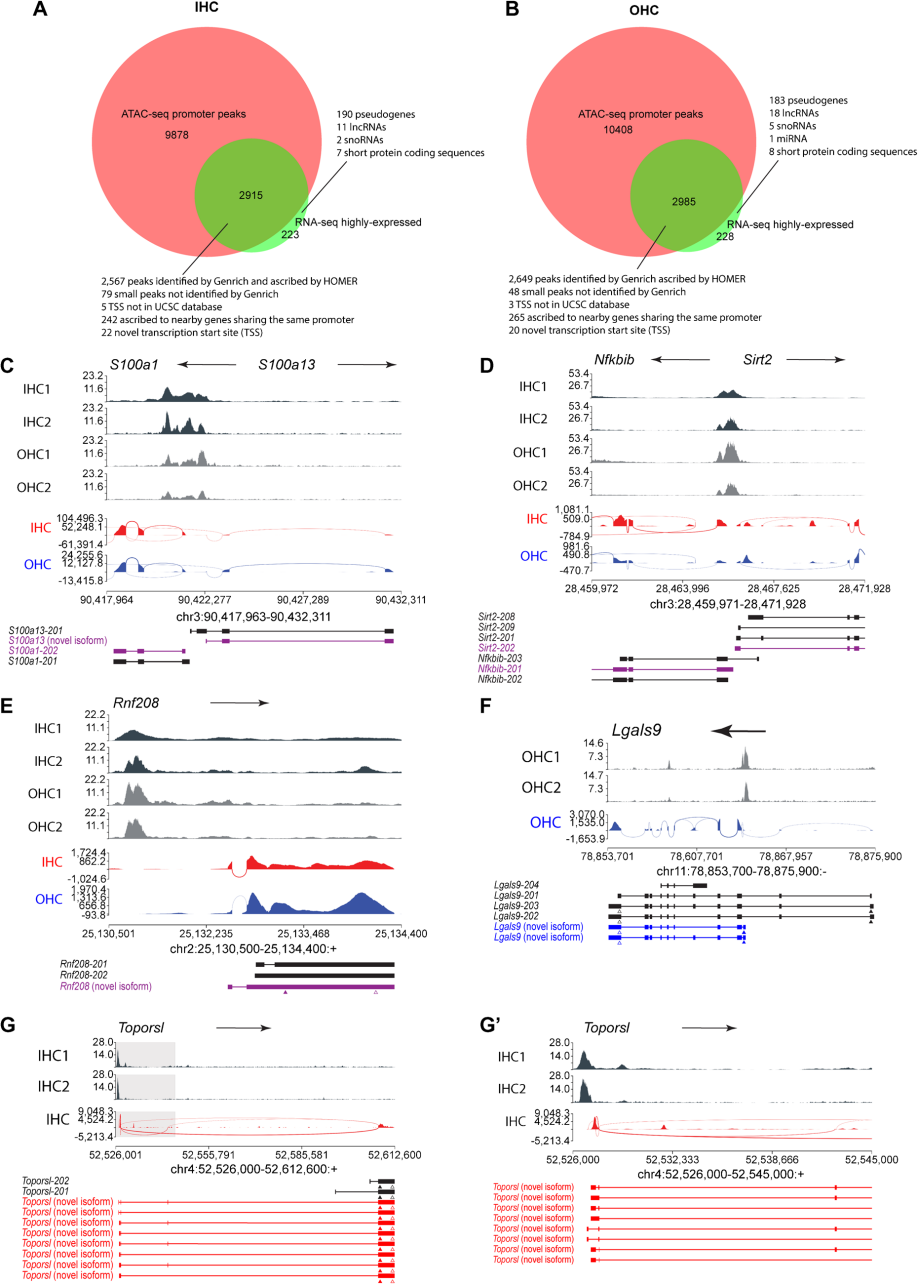
Most genes highly expressed in cochlear hair cells have accessible promoter elements. **A**, **B** Overlap between genes that are highly expressed and genes with an open proximal element in IHCs (**A**) and OHCs (**B**). See Tables S23–S28 for the complete lists of genes. **C** ATAC-seq trace and RNA-seq sashimi plot of *S100a1* and *S100a13*, two genes in opposite directions that have adjacent transcription start sites. Both encode calcium-binding proteins of EF-hand type. Note the ATAC-seq peak in their shared promoter region. **D** ATAC-seq trace and RNA-seq sashimi plot the promoter region of NFKB Inhibitor Beta (*Nfkbib*) and Sirtuin 2 (*Sirt2*), two genes in opposite directions that have adjacent transcription start sites. Note the ATAC-seq peak in their shared promoter region. **E** ATAC-seq trace and RNA-seq sashimi plot of Ring Finger Protein 208 (*Rnf208*), a gene expressed in both IHCs and OHCs. Note the putative novel TSS upstream of canonical TSS. **F** ATAC-seq trace and RNA-seq sashimi plot of Galectin 9 (*Lgals9*), a gene expressed in OHCs but not IHCs. Note the putative novel TSS downstream of canonical TSS. **G**, **G’** ATAC-seq trace and RNA-seq sashimi plot of the full length of Topoisomerase I Binding, Arginine/Serine-Rich Like (*Toporsl*), a gene expressed in IHCs but not OHCs. Note the putative novel TSS upstream of the canonical TSS. Gray box indicates area zoomed in **G’**. For **C**–**G’**, the arrow at the top of each trace indicates the direction of the gene; the ATAC-seq traces are black for IHCs and grey for OHCs; the RNA-seq sashimi plots are colored red for IHCs and blue for OHCs; and below them are depicted the transcripts (published or found in this study) that each gene produces, with those not expressed by HCs in black, those expressed solely by IHCs in red, solely by OHCs in blue, and by both IHCs and OHCs in purple. Arrowheads point to the start (filled) and end (open) of the coding sequences

To test the reliability of our ATAC-seq data, we then looked at the overlap between the genes that are highly expressed in each cell type and genes that have proximal ATAC-seq peaks, as we would expect all genes that are expressed to have open chromatin at their promoters. For this study, we computed TPM values from the RNA-seq data using TPMCalculator [22] and defined genes with a TPM of at least 15 as highly expressed. Many (84.2% in IHCs, 82.2% in OHCs) of these genes are expressed in both IHCs and OHCs, but we analyzed them separately for each cell type, as it provides two independent tests of open promoter for gene expression. Most open promoters corresponded to genes that were not expressed in HCs (Fig. 5A, B). This is not surprising given that there may be a lack of transcription factors or additional chromatin modifications that are needed for these genes to be expressed. The relevant question is whether most genes that are being expressed have an open promoter. Our initial computerized analysis detected proximal peaks in 2568 out of 3145 genes that are highly expressed in IHCs (Fig. 5A, Table S23) and 2650 out of 3220 genes that are highly expressed in OHCs (Fig. 5B, Table S24). However, when we individually examined the 577 highly expressed genes in IHCs for which the software did not identify a proximal peak, we realized that 79 had a small peak not detected by the peak caller Genrich but was clearly discernible by eye, 5 overlapped a transcription start site that was not listed in UCSC Genome Browser’s even though the gene was listed in Ensembl’s database, 242 had been ascribed to a nearby gene sharing the same promoter region, usually going in the opposite direction, some of which are co-expressed with the gene in question, 22 had a novel TSS (seen with a promoter peak near the novel TSS), and only 223 lacked a visible peak, of which 190 are pseudogenes (Fig. 5A, Table S25, S27). Very similar numbers were obtained for OHCs (Fig. 5B, Table S26, S28).

Most of the highly expressed genes whose proximal peak was ascribed to another gene had an adjacent gene going in the opposite direction, thus sharing a common promoter. Examples include *S100a13* and *Nfkbib*, whose promoter peaks had been ascribed by HOMER to *S100a1* and *Sirt2*, respectively (Fig. 5C, D).

Examples of genes that had novel TSS included *Rnf208*, *Lgals9*, and *Toporsl*. In the case of *Rnf208* and *Toporsl*, the novel TSS was upstream of the canonical one, but did not result in any changes to the open reading frame compared to the canonical isoform (Fig. 5E, G, G’). This suggests that although the protein produced is the same, the alternative TSS in HCs might allow its expression to be regulated by a different set of transcription factors from the canonical one in other cell types. *Lgals9*, on the other hand, has a downstream TSS that results in an open reading frame coding for a truncated protein (Fig. 5F). The function of *Lgals9* in HCs has yet to be established, but this isoform might, in addition to or instead of producing a truncated protein, be expressing a non-coding RNA, since many genes with short open reading frames are predicted to be lncRNAs.

The few genes for which no proximal peak was detectable by any of our criteria were mostly pseudogenes, and the remaining ones mostly comprised of 11 long non-coding RNAs (lncRNAs), 2 small nucleolar RNAs (snoRNAs) and 6 short protein coding sequences in IHCs, and 18 lncRNAs, 5 snoRNAs, 1 microRNA (miRNA), and 8 short protein coding sequences in OHCs. Notwithstanding these exceptions, most genes expressed in IHCs (92.9%) or OHCs (92.3%) have accessible (as detected by ATAC-seq) promoter regions in their respective cell types. This confirms that our ATAC-seq data detects most active promoters as open chromatin.

### A Significant Number of Genes Differentially Expressed Between IHCs and OHCs Have Differentially Accessible Promoters and/or Enhancers

**Fig. 6:**
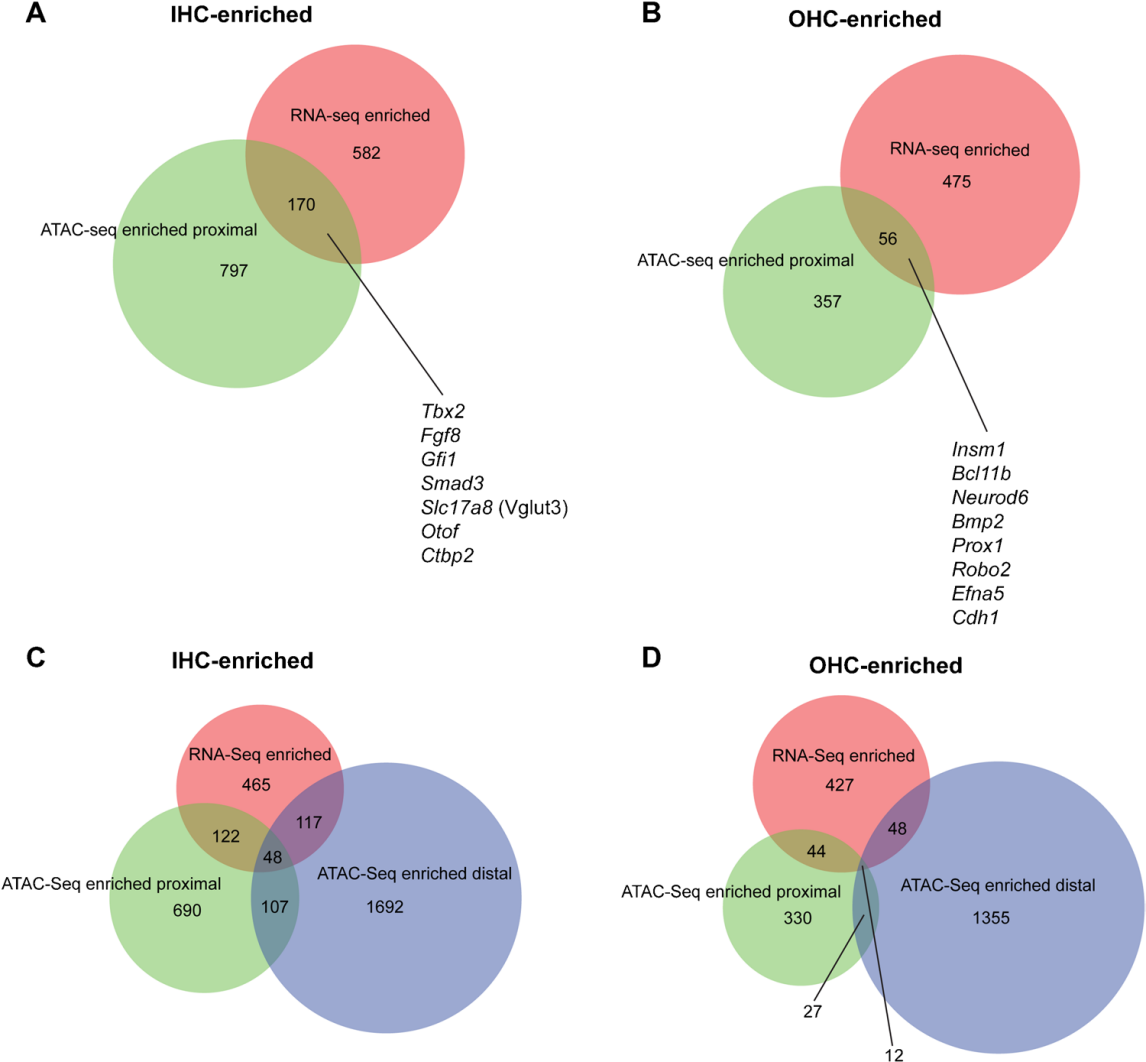
Differential chromatin accessibility corresponds to differential expression in few but prominent IHC –vs OHC–enriched genes. **A** IHC-enriched gene expression vs IHC-enriched proximal elements (*p* = 5.984779e-137, expected overlap at random = 13.1). Listed are examples of functionally relevant genes that are preferentially expressed and have enhanced promoter accessibility in IHCs. See Table S29 for the complete list of genes. **B** OHC-enriched gene expression vs OHC-enriched proximal elements (*p* = 1.976857e-46, expected overlap at random = 3.96). Listed are examples of prominent genes that are preferentially expressed and have enhanced promoter accessibility in OHCs. See Table S31 for the complete list of genes. **C** Three-way comparison of genes with IHC-enriched expression, proximal elements, and distal elements. See Table S30 for the complete list of genes with enriched distal but not proximal elements. **D** Three-way comparison of genes with OHC-enriched expression, proximal elements, and distal elements. See Table S32 for the complete list of genes with enriched distal but not proximal elements

Gene expression can often be regulated by accessibility to the transcription machinery in the promoter region. In order to determine whether differences in gene expression between IHCs and OHCs can be explained at least in part by differential accessibility in the chromatin, we checked whether genes with differentially enriched expression also had differentially accessible proximal elements. We found that out of 752 genes with enriched expression in IHCs, 170 had more accessible proximal elements (many more than the 13.1 that would be expected at random; Fig. 6A, Table S29), while out of 531 genes with enriched expression in OHCs, 56 had more accessible proximal elements (vs 3.96 expected at random; Fig. 6B, Table S30). Of the genes that did not have a more accessible proximal element, 117 IHC-enriched genes (Fig. 6C, Table S31) and 48 OHC-enriched genes (Fig. 6D, Table S32) had more accessible distal elements (potential enhancers) in the respective cell types. A hypergeometric test found that the overlap between the gene lists for enriched expression and more open proximal chromatin elements is statistically significant for both IHCs (*p* = 5.984779e-137) and OHCs (*p* = 1.976857e-46). Although of the differentially expressed genes those with differentially accessible promoters were only a fraction (22.6% for IHCs and 10.5% for OHCs; Fig. 6A, B; a greater percentage is obtained when including potential enhancers), these include most of the genes with known cell type-specific functions, such as *Tbx2*, *Fgf8, Slc17a8* (Vglut3), *Otof*, and *Ctbp2* in IHCs (Fig. 6A) and *Insm1*, *Prox1*, *Efna5*, and *Cdh1* in OHCs (Fig. 6B). Interestingly, this includes functional genes (such as *Slc17a8*/*Vglut3*, *Otof*, and *Ctbp2*) whose expression is incipient in neonatal hair cells but will be maintained throughout life, as well as developmental genes (such as *Tbx2* and *Fgf8* in IHCs and *Insm1* and *Prox1* in OHCs) which are only expressed transiently. Overall, our results reveal that the difference in expression for a significant number of genes in each hair cell type can be explained by the differences in chromatin accessibility of their promoter regions and that this is true for both permanently and transiently expressed genes.

### Some Genes Expressed in Neonatal Hair Cells Have Novel Transcription Start Sites and Generate Cochlear Isoforms Not Reported in Other Organs or Cell Types

**Fig. 7:**
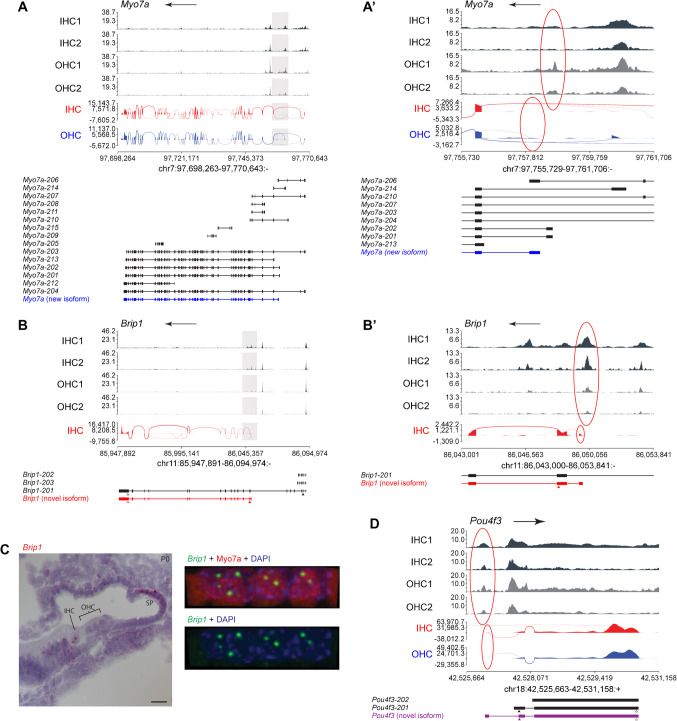
Combined ATAC-seq and RNA-seq can reveal novel isoforms with HC-specific exons and transcription start sites. **A**, **A’** ATAC-seq traces and RNA-seq sashimi plots of *Myo7a*. The full-length gene is shown in **A**, while the area in the box is zoomed in **A’**, with the OHC-specific exon and its adjacent open proximal element circled in red. **B**, **B’** ATAC-seq traces and RNA-seq sashimi plot of *Brip1*. The full-length gene is shown in **B**, while the area in the box is zoomed in in **B’**, with the novel exon and its adjacent open proximal element circled in red. **C** RNA in situ hybridization images of *Brip1* in mice at P0. Scale bar is 20 µm. Structures are labelled with the following abbreviations: IHC, inner hair cells; OHC, outer hair cells; SP, spiral prominence. Image on the left is a cross section of the mouse cochlea in which *Brip1* mRNA is detected with a chromogenic dye. Note how the *Brip1* mRNA seems to localize to the nuclei of the IHC and the cells of the spiral prominence. Images on the right are confocal projections from the top of a row of IHC nuclei in which *Brip1* mRNA is detected with a fluorescent label. About two stronger foci are visible within each IHC nuclei. DAPI counterstain (blue) reveals that *Brip1* mRNAs accumulate in genomic regions of low DNA density (i.e., euchromatin). **D** ATAC-seq traces and RNA-seq sashimi plots of *Pou4f3*, with the novel exon and its adjacent open proximal element circled in red. For **A**–**B’** and **D**, the arrow at the top of each trace indicates the direction of the gene, the ATAC-seq traces are black for IHCs and grey for OHCs, the RNA-seq sashimi plots are colored red for IHCs and blue for OHCs, and below them are depicted the transcripts (published or found in this study) that each gene produces, with those not expressed by HCs in black, those expressed solely by IHCs in red, solely by OHCs in blue, and by both IHCs and OHCs in purple. Arrowheads point to the start (filled) and end (open) of the coding sequences

Some of the searches for promoters of expressed genes had revealed the existence of novel exons, and therefore mRNA isoforms, in hair cells. We therefore used this combinatorial examination of RNA-seq and ATAC-seq to seek novel mRNA isoforms and promoters for other genes. As further proof of concept, we also examined *Myo7a*, a gene that reportedly produces two mRNAs starting in different exons, one exclusively expressed in OHCs but not IHCs [56]. Our RNA-seq data confirms the existence of this isoform in OHCs, and our ATAC-seq data reveals a proximal element near the TSS of the OHC-specific isoform that is accessible only in OHCs (Fig. 7A, A’).

We then inspected some other genes known or suspected to play a role in hair cell development and found novel isoforms of the helicase-encoding *Brip1* and of the transcription factor-encoding *Pou4f3*. As previously reported, *Brip1* expression is enriched in IHCs relative to the OHCs during development at P0 [8]. On closer inspection, we found that the transcript of *Brip1* in IHCs lacks the first six exons and instead begins at a novel TSS within intron 6, which we name exon 6B (Fig. 7B, B’). This novel mRNA isoform of *Brip1* lacks in its open reading frame the coding sequence for the nuclear localization signal (NSL) and part of the helicase domain. We find a region of open chromatin proximal to the novel exon 6B, which is significantly more accessible in IHCs than OHCs and hence qualifies as a putative novel promoter. RNA in situ hybridization suggests that the *Brip1* transcript is localized to the nucleus instead of the cytoplasm in IHCs. Within the nucleus, it is concentrated in discrete foci of low DNA density (i.e., euchromatin; Fig. 7C). This subcellular pattern may suggest that in IHCs, *Brip1* produces a non-coding RNA rather than a protein-coding transcript.

*Pou4f3*, on the other hand, exhibits a previously uncharacterized exon upstream of the canonical first exon, with a region of open chromatin (presumably the promoter) just upstream of the novel exon (Fig. 7D). This new mRNA isoform of *Pou4f3* shares the same open reading frame with the canonical mRNA, as it does not contain an upstream start codon. Hence, the two *Pou4f3* mRNA isoforms differ only in the 5′ untranslated region (UTR). The functional reasons for this are therefore not production of a functionally distinct POU4F3 protein isoform but are perhaps regulatory. For example, by using an alternative promoter, *Pou4f3* might be regulated in hair cells by a different set of transcription factors from those that regulate the production of the canonical mRNA in other cell types.

These examples provide proof of principle for how our combined RNA-seq and ATAC-seq datasets may be used to identify novel transcription start sites (new promoter + first exon combinations). While we could not systematically examine all genes, our datasets provide the resource for the individual examination of any gene of interest.

## Discussion

Here, we first extended the RNA-seq data analysis of developing cochlear IHCs and OHCs to look at alternative RNA splicing as a factor in their different transcriptomes. While single-cell RNA-seq allows for the transcription levels of individual genes to be quantified in each cell type, it cannot inform on splice isoforms as it uses short-read sequencing at the 3′ ends of the transcripts. However, using bulk RNA-seq data from pools of IHCs and OHCs, we were able to determine that in addition to differences in gene expression, there were also differences in splicing of their expressed transcripts. This suggests that in addition to regulation of gene expression, regulation of RNA splicing also plays a role in the differential development of IHCs and OHCs.

Future studies could further validate differential exon usage between IHCs and OHCs for any gene of interest using methods such as mRNA in situ hybridization for alternatively spliced exons or TaqMan quantitative reverse transcription polymerase chain reaction (RT-qPCR) using exon-specific internal probes. Functional studies of these differentially spliced exons could be performed by isoform-specific mutagenesis.

Some of the alternative splicing we have observed results in protein-coding transcripts becoming non-coding RNAs instead. Some of these alternatively spliced exons may also potentially function as poison exons [57], which play a role in regulating protein expression by introducing a premature stop codon to the mRNA that results in nonsense-mediated mRNA decay. This may represent a posttranscriptional mechanism for preventing protein expression or may reveal non-protein roles for these non-coding RNAs during IHC and OHC differentiation.

Differential gene expression can often be explained by differences in the accessibility of chromatin in proximal (i.e., promoter) or distal (e.g., enhancer) regulatory elements, as more open chromatin makes these genomic regions more accessible to the transcriptional machinery. Most differentially expressed genes did not exhibit significant differences in accessibility (as determined by ATAC-seq) of their promoters (proximal elements) between IHCs and OHCs, which is not surprising, as transcription can also be regulated by other means, such as DNA and histone modifications or binding of specific transcription factors. However, a significant number of differentially expressed genes had a significant difference in accessibility of chromatin in their promoters. These include genes previously found to play major roles in the separate development of IHC and OHC, such as *Tbx2* [9] and *Fgf8* [58] in IHCs and *Insm1* [8] and *Efna5* [32] in OHCs. Interestingly, we also found that *Gfi1*, a gene required for both IHC and OHC differentiation [4], is enriched about twofold in IHCs relative to OHCs, and also has a more accessible promoter (proximal element) in IHCs than OHCs. This suggests that the expression level of GFI1 might play a role in determining developmental differences between IHCs and OHCs.

By combining RNA-seq and ATAC-seq data, we were able to identify a few genes with novel promoters for HCs that were not previously described in other cell types. Some of these had novel exons upstream of the canonical TSS that extended the 5′ UTR but did not result in any changes to the open reading frame. This suggests that the regulation of gene expression may also occur by using alternative promoters to drive production of the same protein in different cell types, which would allow for transcription to be initiated using different transcription factors. We also found that *Brip1*, which canonically encodes a helicase, was expressed in a form whose open reading frame appears to result in a non-functional helicase protein, and whose RNA transcript appears to localize to discrete clouds in the euchromatin (active chromatin) part of the nucleus. A similar-looking type of RNA “cloud” has previously been reported for *Evf2*, a long non-coding RNA that accumulates at its site of transcription, over an enhancer, from which it regulates nearby genes [59]. It is intriguing that the *Brip1* locus is very close to that of *Tbx2* (~ 200 kb apart, with *Tbx4* as the sole intervening gene), and that both genes are co-expressed in IHCs and other cells of the inner compartment. An obvious suggestion is that of a regulatory interaction between *Brip1* and *Tbx2*. The case of *Brip1* illustrates that, as for alternative splicing, alternative promoters and starting exons (and Transcription Start Sites) in IHCs and OHCs may determine whether each cell type expresses a protein-coding form and a non-coding RNA for some genes.

We found smaller ATAC-seq peaks that were visible by eye but were not identified by the peak caller Genrich. This is likely because peak callers use statistical methods to identify regions of open chromatin, and smaller peaks may sometimes not exceed the threshold above background to be positively identified. However, the fact that the gene is expressed at a reasonably high level suggests that these peaks are nevertheless real. New bioinformatics tools that take RNA-seq data into account would thus be useful for the identification of regions of open chromatin that would be missed by current peak callers.

Although much progress has been made in compiling databases of transcripts in multiple mouse cell types and tissues, we discovered that some of the transcript variants present in HCs are novel, not present in pre-existing databases and hence in other cell types. This is consistent with the unique features and development of HCs. In this study, we identified numerous genes with novel TSS by manually examining expressed genes that did not have a region of open chromatin in their canonical promoter regions. However, this approach would miss genes whose canonical promoter regions are accessible but that are expressed from novel TSS (e.g., *Brip1* and *Pou4f3*). Identification of novel isoforms, and even of new genes, systematically on a global scale will require development of bioinformatics tools that combine RNA-seq and ATAC-seq.

As a resource, our data reveals for each gene which mRNA isoforms are expressed in IHCs and/or OHCs, whether novel or previously found in other cell types. While we only describe in some detail the mRNA isoforms and novel proximal regulatory elements (promoters) for a few genes, our datasets are available for any willing investigator to examine and visualize with free software, such as Integrated Genomics Viewer (IGV), the state of their gene, and transcript of interest in IHCs and OHCs. This will be useful in several ways: first, to design or use the appropriate gene-targeting strategies. As an example, we ruled out the use of two available presumed KOs of *Brip1* because they either deleted exon 3 or introduced a gene trap (splice acceptor followed by poly-adenylation signal) in intron 5, both upstream of the *Brip1* proximal regulatory element and mRNA isoform that IHCs express. Prior to engineering or simply importing mice for gene targeting in hair cells, the database provided here should be examined. Second, for detection procedures of any gene of interest, the mRNA isoform expressed in hair cells should be examined to ensure that it complements the probe to be used for in situ hybridization or encodes the epitope detected by the antibody to be used in immunodetection. For example, the first commercially available in situ hybridization probe for *Bcl11b* complemented a predicted 3′ UTR not included in the mRNA expressed in OHCs, and hence we designed an appropriate probe. Third, the promoters and potential enhancers should be of much utility for gene therapy. We reveal numerous promoter fragments (open chromatin peaks) that might drive gene expression exclusively in hair cells but not in other cochlear cell types (e.g., *Pou4f3*; Figs. 4D and 7D), solely IHCs (e.g., *Fgf8* during development and *Slc17a8/Vglut3* throughout life; Fig. 4F, G), solely OHCs (e.g., *Insm1* or *Neurod6* during development and the internal *Myo7a* promoter throughout life; Figs. 4H, I and 7A’), or either type of hair cells in combination with other cochlear cell types, such as perhaps *Cdh1* for the entire outer compartment hair and supporting cells, the internal promoter of *Brip1* for IHCs and other Greater Epithelial Ridge cells (Fig. 7B’), and *Tbx2* for IHCs and most of the epithelium except those in the outer compartment (Fig. 4E). Finally, by revealing non-coding sequences either expressed in mRNAs or accessible for potential use as promoters and enhancers, genetic studies can identify whether single nucleotide polymorphisms (SNPs) and other candidate mutations not disrupting coding sequences but associated with hearing loss might be causative.

### Limitations of the Study

While we identified numerous putative novel promoters of known genes, this study is unable to identify the precise TSS for the novel isoforms. This could be achieved by 5′ rapid amplification of cDNA ends (RACE). Moreover, these putative promoters for IHCs or OHCs will need validation by a combination of promoter mutagenesis and transgenic or viral vector-mediated reporter expression.

Although we identified potential enhancers by ascribing distal ATAC-seq peaks to the nearest gene, this method is not foolproof as enhancers do not necessarily regulate the nearest gene, and some regions of high chromatin accessibility may be other types of regulatory elements such as insulators [60] or silencers [61, 62]. To know for certain that a distal element is interacting with the promoter of a specific gene, we would need 3D chromatin conformation data obtained with technologies such as high-throughput chromosome conformation capture (Hi-C) [63, 64]. Furthermore, mutagenesis experiments of the distal element would be required to demonstrate effects of a putative enhancer disruption on the expression level of its candidate gene. Unfortunately, as Hi-C and related technologies currently require over 20 million cells, the limited number of hair cells per animal renders this approach unfeasible at present.

Moreover, while it is possible to look at the relative abundance of splice events at individual splice junctions using our RNA-seq data, assembling whole transcript isoforms is more challenging using Illumina’s high-throughput sequencing systems due to the short length of its reads. Hence, for genes subjected to alternative splicing at separate locations, our data cannot determine the mRNA isoforms being expressed. This could be resolved with long-read RNA-seq technologies (e.g., Iso-Seq by Pacific Biosciences or SMART-Seq by Takara).

## Materials and Methods

### Animals

All experiments were conducted in accordance with the policies of the Institutional Animal Care and Use Committee (IACUC) of Northwestern University. Timed pregnancies were set up by putting male and female mice in the same cage in the evening, and separating them the next morning, with the day of separation being designated as E0. The day of birth was designated as postnatal day P0.

Animal experiments were planned in accordance with the ARRIVE guidelines. From each P0 pup, IHCs and OHCs were collected in separate pools for ATAC-seq Library synthesis. Two separate pools, each of IHCs and OHCs, were collected. Each pool consisted of IHCs or OHCs from 9 pups. All animals were wildtypes as we were comparing the chromatin states of two different cell types, and both cell types were collected from each pup. The individual pups were not sexed as each pool of cells came from multiple animals.

### Fluorescence-activated cell sorting (FACS)

For fluorescence-activated cell sorting (FACS), IHCs and OHCs were labelled using a method previously used by Wiwatpanit et al*.* [8], in which homozygous B6.Cg-Gt(ROSA)26Sortm9(CAG-tdTomato)Hze/J males (JAX #:007909; RRID: IMSR_JAX:007909) were mated with hybrid Insm1tm1.1(GFP/cre)Mgn/Mmjax (JAX #036986; RRID: MMRRC_036986-JAX) and B6.129S-Atoh1tm4.1Hzo/J (JAX #013593; RRID: IMSR_JAX:013593) females, so the IHCs would be labelled by GFP and the OHCs would be labelled by both GFP and TdTomato at P0. Cells were sorted into a solution of 2% fetal bovine serum (FBS, Gibco #A38402-01) in 1 × PBS using a BD FACSARIA flow cytometer.

### ATAC-seq

After FACS, ATAC-seq libraries were synthesized using a custom-made Tn5 transposase from the Neil Segil lab at the University of Southern California (USC) as described by Tao et al. [53]. Cells in separate IHC and OHC pools were centrifuged at 700 g for 20 min at 4 °C. The supernatant was discarded, and 20 μl cold lysis buffer (10 mM Tris–HCl pH 8.0, 5 mM MgCl_2_, 10% DMF, 0.2% NP-40) was added to the pellet. 20 μl reaction buffer (10 mM Tris–HCl pH 8.0, 5 mM MgCl_2_, 10% DMF, 3000 units Tn5 transposase) was added to the lysis buffer, and after mixing well, the reaction mix was incubated at 37 °C for 30 min. The fragments were then purified using the Qiagen MinElute Reaction Cleanup Kit (Qiagen #: 28,204).

The Library was then synthesized using PCR, with an initial 5-cycle PCR performed with the using the following mix: 23 μl purified DNA fragments after Tn5 transposase, 2.5 μl Primer 1 (25 μM), 2.5 μl Primer 2 (25 μM) with barcorde, 0.3 μl SYBR green (Invitrogen #: S7563) and 25 μl NEBNext® High-Fidelity 2X PCR Master Mix (NEB #: M0541S). SYBR green was diluted to 100X in DMSO prior to addition to PCR reaction mix. Primer sequences are listed in Table 3, with a common forward primer for all samples, and differing reverse primers giving them different barcodes. The initial 5-cycle PCR was performed using the following conditions:
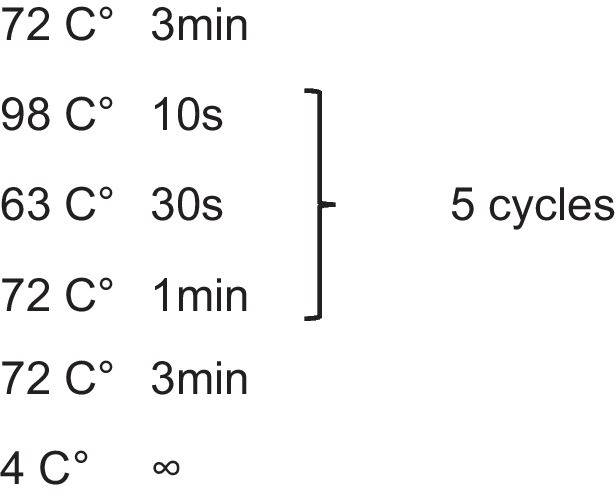

**Table 3:** Sequences of primers used for library synthesis in ATAC-seq and i7 index barcodes

Primer	Sequence	Barcode
Forward	AATGATACGGCGACCACCGAGATCTACACTCGTCGGCAGCGTCAGATGTG	N/A
IHC1 Reverse	CAAGCAGAAGACGGCATACGAGATTTCTGCCTGTCTCGTGGGCTCGGAGATGT	AGGCAGAA
IHC2 Reverse	CAAGCAGAAGACGGCATACGAGATAACCCCTCGTCTCGTGGGCTCGGAGATGT	GAGGGGTT
OHC1 Reverse	CAAGCAGAAGACGGCATACGAGATGCTCAGGAGTCTCGTGGGCTCGGAGATGT	TCCTGAGC
OHC2 Reverse	CAAGCAGAAGACGGCATACGAGATCCCAACCTGTCTCGTGGGCTCGGAGATGT	AGGTTGGG

5 μl of each PCR reaction mix was taken for qPCR to determine the number of cycles needed to reach saturation point. This was mixed with 5 μl UltraPure distilled water (Invitrogen #: 10,977–035) and 5 μl NEBNext® High-Fidelity 2X PCR Master Mix to make up a final volume of 15 μl. qPCR was performed using the QuantStudio 7 Flex Real-Time PCR System (Applied Biosystems #: 4,485,701) using the following cycling conditions:


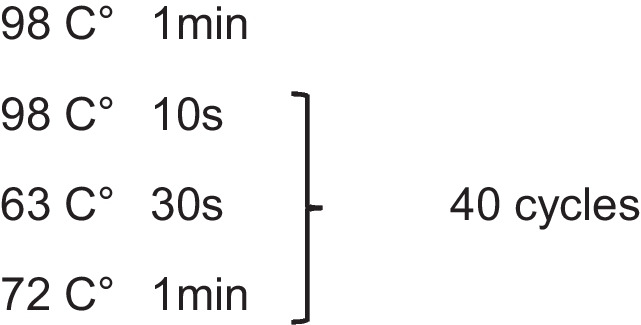


Additional cycles were then performed on the rest of the PCR reaction mix with to the following cycling conditions, with n being the number of cycles needed to reach ¼ saturation point as determined from the aforementioned qPCR step:


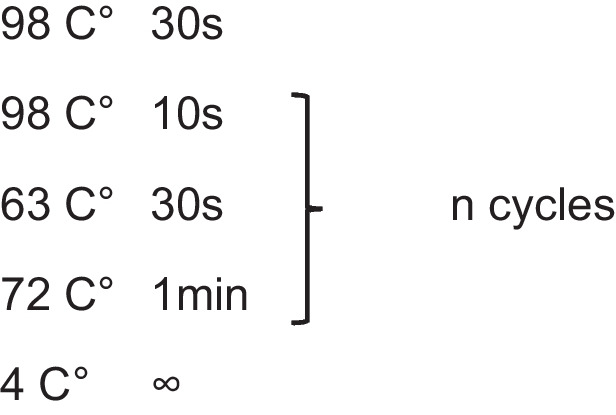


45 AMPure XP PCR Purification Reagent (Beckman Coulter #: A63880) was mixed with the PCR product to bind Library fragments to SPRI magnetic beads. The mix was incubated on a magnetic stand for 2 min, and the supernatant was discarded. Beads were washed twice in 200 μl 80% ethanol for about 2 min each time. DNA fragments were eluted from the beads using 22 μl UltraPure distilled water and incubating at room temperature for 5 min.

ATAC-seq Libraries were sent to the NUSeq Core Facility at Northwestern University for fragment size analysis using the BioAnalyzer system, as well as size selection for fragments from 150 and 1000 bp in size. Libraries were then sequenced using the Illumina NextSeq 500 system by the NUSeq Core Facility with 37 bp paired-end reads to a depth of 50 million reads per sample. IHCs and OHCs from multiple embryos were pooled together to generate ATAC-seq Libraries with 2 biological replicates for each cell type.

### Bioinformatics

For all bioinformatics analyses mentioned in this section, the default settings were used unless otherwise indicated.

ATAC-seq reads were aligned to the mouse genome using Bowtie2 (RRID: SCR_016368) [49], with the “–very-sensitive” and “–no-mixed” options turned on, and a maximum fragment length (-X) of 1000. For this study, we used the GRCm39 version of the mouse genome using the vM27 annotation from GENCODE (RRID: SCR_014966) [66] as our reference. Alignment files were sorted and indexed using SAMtools (RRID: SCR_002105) [67]. ATAC-seq peaks were identified using Genrich (RRID: SCR_025320) [51] in ATAC-Seq mode with PCR duplicate removal and a FDR-adjusted p-value of 0.05 as the threshold. IHC and OHC peaks were called separately, then sorted and merged into a single peak file using BEDTools merge (RRID: SCR_006646) [55]. Peaks were annotated and the number of reads under each peak for each sample was quantified using HOMER’s annotatePeaks.pl module, and BigWig trace files were generated using HOMER’s makeUCSCfile module (RRID: SCR_010881) [52]. Differential ATAC-seq peak enrichment was computed using edgeR (RRID: SCR_012802) [16–19]. ATAC-seq heat maps were generated using Deeptools (RRID: SCR_016366) [68]. For the HC vs SC analysis, ATAC-seq data from Gene Expression Omnibus (GEO, RRID: SCR_005012) (Accession no.: GSE150010). The overlap between HC vs SC-enriched peaks, and IHC vs OHC-enriched peaks was determined used the BEDTools intersect function.

RNA-seq reads were aligned to the mouse genome using STAR (RRID: SCR_004463) [13]. A first pass through star was run to generate a list of splice junctions. This list was then fed back to STAR in a second pass using the “–sjdbFileChrStartEnd” option to allow for more sensitive mapping of reads to novel splice junctions. Read counts for each gene were quantified with HTSeq (RRID: SCR_011867) using the intersection-nonempty quantification mode [14]. Differential gene expression was computed using edgeR [16–19]. For the HC vs non-HC analysis, RNA-seq data from Cai et al*.* was downloaded from GEO (Accession no.: GSE65633).

ATAC-seq and RNA-seq alignments were visualized using Integrative Genomics Viewer (IGV. RRID: SCR_011793) [69–72]. Sashimi plot and ATAC-seq trace images for figures were plotted using Trackplot [73]. Venn diagrams were plotted using BioVenn [74].

### RNA in situ Hybridization and Immunohistochemistry

Inner ears were prepared for sectioning by fixing in 4% (w/v) paraformaldehyde (PFA) in 1 × phosphate-buffered saline (PBS) for 24 h at 4 °C following dissection. Inner ears were cryopreserved by passing them through a series of sucrose solutions in 1 × PBS of increasing concentration for 1 h at 4 °C each, of 5%, 10% and 20% in concentration respectively. Inner ears were then incubated for 24 h at 4 °C in a mixture of 50% Tissue-Tek O.C.T. Compound (Sakura #4583) and 10% sucrose in 1 × PBS. Finally, tissues were frozen in Tissue-Tek O.C.T. Compound at −80 °C for cryosectioning.

RNA in-situ hybridization on cochlear sections was performed using the RNAscope 2.5 HD Assay – RED (ACD Bio #322,350), and nuclei were labelled using haematoxylin for the bright field images. For the fluorescent images, nuclei were labelled using DAPI (Invitrogen #D21490), and hair cells were labelled using rabbit anti-Myo7a primary antibody (Proteus Biosciences #25–6790; RRID: AB_10015251) and AlexaFluor 488 donkey anti-rabbit secondary antibody (Jackson Immunoresearch #711–545-152; RRID: AB_2313584). Images were taken using a Nikon Eclipse E600 microscope.

Fluorescent RNA in-situ hybridization for *Brip1* on whole mount organ of Corti was performed using the RNAscope Multiplex Fluorescent V2 Assay (ACD Bio # 323,110) with Opal 520 (Akoya Biosciences #FP1487001KT) fluorophore. Hair cells were labelled using rabbit anti-Myo7a primary antibody and AlexaFluor 594 donkey anti-rabbit secondary antibody (Jackson Immunoresearch #711–585-152; RRID: AB_2340621), and nuclei were labelled using DAPI. Confocal microscopy was performed at the Center for Advanced Microscopy at Northwestern University using a Nikon A1R confocal microscope.

### Statistical Analysis

To determine whether the overlap between two gene sets was significant, we performed a hypergeometric test. The p-value was calculated using the cumulative distribution function of the hypergeometric distribution, in which $P\left(x\ge k\right)$ is the probability of observing *k* or more genes in common between the two sets had they been selected at random, which is as follows:

$$\text{P}\left(x\ge k\right)=\sum\limits_{i=k}^{\text{min}\left({n}_{A},{n}_{B}\right)}\frac{\left(\genfrac{}{}{0pt}{}{{n}_{A}}{i}\right)\left(\genfrac{}{}{0pt}{}{N-{n}_{A}}{{n}_{B}-i}\right)}{\left(\genfrac{}{}{0pt}{}{N}{{n}_{B}}\right)}$$

where *N* is the total number of genes in the mouse genome (55,416 in the vM27 annotation), ${n}_{A}$ is the number of genes in set A, ${n}_{B}$ is the number of genes in set B and *k* is the number of genes in common between the two sets. $\left(\genfrac{}{}{0pt}{}{a}{b}\right)$ is the binomial coefficient $a$ choose $b$, and $\text{min}\left({n}_{A},{n}_{B}\right)$ is the lesser of the two values between ${n}_{A}$ and ${n}_{B}$, representing the maximum possible overlap between the two gene sets. We considered the number of genes in common to be significant if the p-value was less than 0.05.

The expectation value of the hypergeometric distribution $\text{E}\left[k\right]$, representing the number of genes in common between the two sets we would expect to see had they been selected at random, was computed using the following equation:

$$\text{E}\left[k\right]=\frac{{n}_{A}\cdot {n}_{B}}{N}$$

## Supplementary Information

Below is the link to the electronic supplementary material.

Table S1−S2 (XLSX 425 KB)

Table S3 (XLSX 6.23 MB)

Table S4−S5 (XLSX 262 KB)

Table S6−S7 (XLSX 182 KB)

Table S8−S12 (XLSX 486 KB)

Table S13−S14 (XLSX 1.03 MB)

Table S15−S16 (XLSX 818 KB)

Table S17−S18 (XLSX 507 KB)

Table S19−S20 (XLSX 268 KB)

Table S21−S22 (XLSX 120 KB)

Table S23−S28 (XLSX 1.03 MB)

Table S29−S32 (XLSX 142 KB)

## Data Availability

The datasets generated during this study are available at NCBI Gene Expression Omnibus (NCBI GEO GSE288375 for RNA-seq data and GSE288376 for ATAC-seq data). The ATAC-seq and RNA-seq data can be concurrently viewed for any gene of interest in an IGV console publicly accessible from the Añoveros lab website (https://igvviewer.s3.us-east-2.amazonaws.com/index.html).
